# Learning speech recognition from songbirds

**DOI:** 10.1186/1471-2202-14-S1-P210

**Published:** 2013-07-08

**Authors:** Izzet B Yildiz, Katharina von Kriegstein, Stefan J Kiebel

**Affiliations:** 1 Max Planck Institute for Human Cognitive and Brain Sciences, Leipzig, 04103 Germany; 2 Biomagnetic Center, Hans Berger Clinic for Neurology, University Hospital Jena, Friedrich-Schiller-University Jena, 07747, Germany

## 

Our knowledge about the computational mechanisms underlying human learning and recognition of speech is still very limited [1]. One difficulty in deciphering the exact means by which humans recognize speech is that there are scarce experimental findings at a neuronal, microscopic level. Here, we show that our neuronal-computational understanding of speech learning and recognition may be vastly improved by looking at a different species, i.e., the songbird, which faces the same challenge as humans: to learn and decode complex auditory input partitioned into sequences of syllables, in an online fashion [2]. Motivated by striking similarities between the human and songbird neural recognition systems at the macroscopic level [3,4], we assumed that the human brain uses the same computational principles at a microscopic level and translated a birdsong model [5] into a human speech learning and recognition model. The model performs a Bayesian version of dynamical, predictive coding [6] based on an internal generative model of how speech dynamics are produced. This generative model consists of a two-level hierarchy of recurrent neural networks similar to the song production hierarchy of songbirds [7]. In this predictive coding scheme, predictions about the future trajectory of the speech stimulus are dynamically formed based on a learned repertoire and the ongoing stimulus. The hierarchical inference uses top-down and bottom-up messages, which aim to minimize an error signal, the so-called prediction error.

We show that the resulting neurobiologically plausible model can learn words rapidly and recognize them robustly, even in adverse conditions. Also, the model is capable of dealing with variations in speech rate and competition by multiple speakers. In addition, we show that recognition can be performed even when words are spoken by different speakers and with different accents--an everyday situation in which current state-of-the-art speech recognition models often fail. We use the model to provide computational explanations for inter-individual differences in accent adaptation, as well as age of acquisition effects in second language learning. For the latter, we qualitatively modeled behavioral results from an experimental study [8].
